# Diagnostic Value of Soluble Urokinase-Type Plasminogen Activator Receptor in Addition to High-Sensitivity Troponin I in Early Diagnosis of Acute Myocardial Infarction

**DOI:** 10.3390/biom9030108

**Published:** 2019-03-18

**Authors:** Nils A. Sörensen, Günay Dönmez, Johannes T. Neumann, Julius Nikorowitsch, Nicole Rübsamen, Stefan Blankenberg, Dirk Westermann, Tanja Zeller, Mahir Karakas

**Affiliations:** 1 Department of General and Interventional Cardiology, University Heart Center, 20246 Hamburg, Germany; n.soerensen@uke.de (N.A.S.); guenay.doenmez@gmx.de (G.D.); j.neumann@uke.de (J.T.N.); j.nikorowitsch@uke.de (J.N.); n.ruebsamen@uke.de (N.R.); s.blankenberg@uke.de (S.B.); d.westermann@uke.de (D.W.); t.zeller@uke.de (T.Z.); 2 German Center for Cardiovascular Research (DZHK), Partner Site Hamburg, Lübeck, Kiel, Hamburg, 20246 Hamburg, Germany

**Keywords:** suPAR, troponin, diagnosis, acute coronary syndrome

## Abstract

The soluble urokinase-type plasminogen activator receptor (suPAR) is a new marker for immune activation and inflammation and may provide diagnostic value on top of established biomarkers in patients with suspected acute myocardial infarction (AMI). Here, we evaluate the diagnostic potential of suPAR levels on top of high-sensitivity troponin I (hs-TnI) in a cohort of patients with suspected AMI. A total of 1220 patients presenting to the emergency department with suspected AMI were included, of whom 245 were diagnosed with AMI. Median suPAR levels at admission were elevated in subjects with AMI compared to non-AMI (3.8 ng/mL vs. 3.3 ng/mL, *p* = 0.001). In C-statistics, the area under the curve (AUC) regarding the diagnosis of AMI was low (0.57 at an optimized cut-off of 3.7 ng/mL). Moreover, baseline suPAR levels on top of troponin values at admission and hour 1 reduced the number of patients who were correctly ruled-out as non-AMI, and who were correctly ruled-in as AMI. Our study shows that circulating levels of suPAR on top of high-sensitivity troponin I do not improve the early diagnosis of AMI.

## 1. Introduction

The uPAR (urokinase plasminogen activator receptor) acts as receptor for urokinase (uPA) that itself splits inactive plasminogen into active plasmin [1]. Beyond this function, by interaction with other proteins, uPAR also plays a role in important atherosclerotic cell processes like migration, adhesion, angiogenesis, proliferation, and chemotaxis [2,3]. The circulating biomarker suPAR (soluble urokinase plasminogen activator receptor) is the soluble form of the cell membrane-bound protein uPAR [4]. It is produced by cleavage of membrane-bound uPAR and can be detected in plasma and serum [5]. The suPAR blood level is stable with no diurnal variation and no changes following fasting [6]. It is discussed as a new marker for immune activation and inflammation—pathways, which are important in atherogenesis [7]. Moreover, it has been shown that suPAR is expressed in a variety of cells, which play a critical role in all stages of atherogenesis—from initiation of fatty streaks to progression of atherosclerosis and plaque destabilization [8]. Although results from these experimental studies are fairly consistent, equivocal data have been reported from clinical and epidemiological studies [9]. Several studies investigating the association between circulating levels of suPAR and prognosis in patients with heart failure and coronary heart disease (CHD) have reported fairly strong associations [10]; however, in acute coronary syndrome (ACS) subjects, the results have been less clear [11,12]. Although a recent study from Denmark in 194 patients indicated that suPAR levels are elevated in acute myocardial infarction (AMI) compared to controls, there was no clear evidence of incremental diagnostic value on top of high-sensitivity troponin [12].

Here, we evaluate the diagnostic potential of suPAR levels on top of high-sensitivity troponin I (hs-TnI) in a large cohort of patients with suspected AMI.

## 2. Materials and Methods

### 2.1. Study Population

The BACC (Biomarkers in Acute Cardiac Care) study has been described previously [13,14,15,16,17]. Briefly, we included 1641 patients presenting to the emergency department of the University Heart Center Hamburg with suspected ACS. We excluded subjects with missing suPAR (*n* = 354) and/or hs-TnI (*n* = 23) measurements, and 78 subjects with ST-elevation myocardial infarction (STEMI) (because biomarker measurements are not relevant for this diagnosis—i.e., AMI denotes non-ST-elevation myocardial infarction (NSTEMI) in this report), resulting in a cohort of 1220 patients. All patients were enrolled between July 2013 and March 2016. The inclusion criteria were suspected ACS, age > 18 years, and the ability to provide written informed consent. All patients underwent a routine clinical assessment as described in the current European Society of Cardiology guidelines [17]. Blood was drawn directly at admission and after 3 h. A primary diagnosis of AMI was adjudicated according to current guidelines based on a high-sensitivity troponin T assay (Elecsys; Roche Diagnostics) [13]. The final discharge diagnosis used for the index event was additionally based on all available clinical, laboratory, and imaging findings in the course of the hospital stay. Two cardiologists adjudicated the diagnosis independently. If the adjudicators disagreed about the diagnosis, a third cardiologist refereed. Moreover, non-ST-elevation myocardial infarction (NSTEMI) was diagnosed based on the third universal definition of myocardial infarction [13].

The BACC study was registered at https://www.clinicaltrials.gov (unique identifier: NCT02355457). The Ethics Committee of the medical association of Hamburg/Germany approved the study, which followed the Declaration of Helsinki. All subjects gave written informed consent.

### 2.2. Laboratory Methods

Blood samples were stored at −80 °C under standardized conditions. Routine laboratory parameters were determined at the University Hospital of Hamburg. Hs-TnI was determined from blood samples collected at admission, after 1 and 3 h. The sensitivity of the hs-TnI immunoassay (Abbott Diagnostics, Wiesbaden, Germany, ARCHITECT i1000SR) had a limit of detection at 1.9 ng/L (range 0–50,000 ng/L) and a 10 percent coefficient of variation at a concentration of 5.2 ng/L. The assay-specific 99th percentile was described at 27 ng/L in the general population [13]. The levels of suPAR were detected using the suPARnostic standard enzyme-linked immunosorbent assay (ELISA, ViroGates, Birkerød, Denmark) in frozen plasma samples. The intra-assay variation was 2.75% and the inter-assay variation 9.17%. This is better than reported with previous enzyme linked immunosorbent assay (ELISA)—like the one used by Stephens and colleagues, who reported an intra-assay variation of 6.5%, and an inter-assay variation of 14% [18].

### 2.3. Statistical Methods

The study population was described with respect to various sociodemographic and medical characteristics. Baseline characteristics are presented as median and interquartile ranges (IQRs) for continuous variables and as counts and percentages for dichotomous variables. The accuracy of the different models to assess accuracy of rule-out and rule-in were estimated—for the binary tests, sensitivity, specificity, negative predictive value (NPV), and positive predictive value (PPV) were computed. Receiver operating characteristic curves were produced for suPAR levels measured on admission. All computations were performed with R version 3.3.3 (http://www.r-project.org/) [19]. A *p*-value of <0.05 was considered statistically significant.

## 3. Results

The baseline characteristics of the study participants are presented in Table 1. Of 1220 patients, 975 were diagnosed as non-AMI and 245 as AMI. The mean age of non-AMI patients was 63 years, while it was 70 years in AMI patients. The majority of patients was of male sex (63.1% in non-AMI and 64.9% in AMI patients). Both, hs-TnI (5.1 [2.5, 10.3] vs. 71.7 [16.2, 695.0] ng/L; *p* < 0.001) and suPAR (3.3 [2.3, 4.7] vs. 3.8 [2.6, 5.4] ng/mL; *p* = 0.001) levels at baseline were higher in patients with AMI. Furthermore, patients presenting with AMI more often reported arterial hypertension and hyperlipoproteinemia. A history of coronary artery disease (CAD) was present in 43.7% of AMI patients—among non-AMI patients still 31.8% reported a history of CAD.

**Figure 1:**
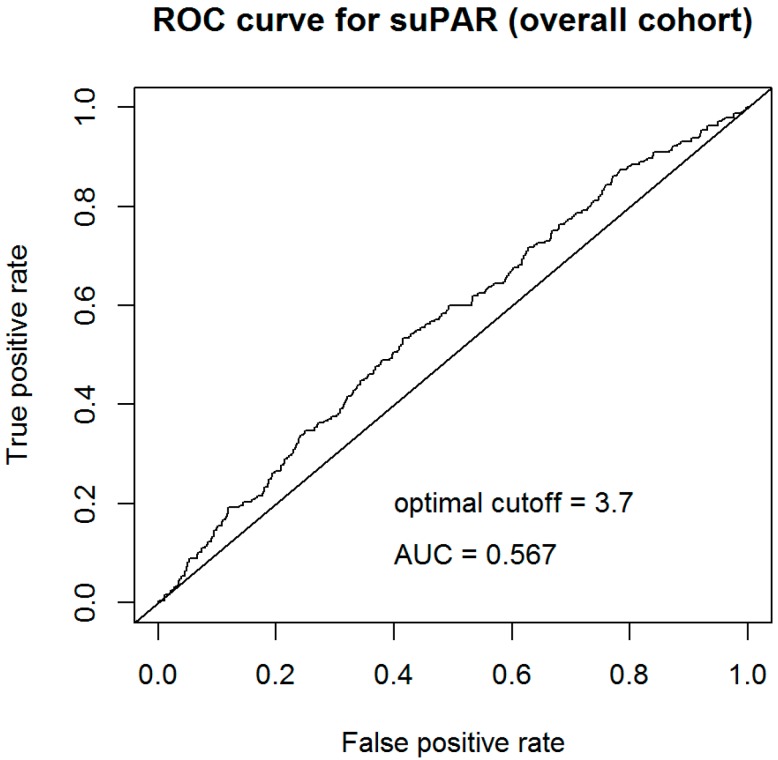
C-statistic for the prediction of acute myocardial infarction (AMI) using baseline levels of soluble urokinase-type plasminogen activator receptor (suPAR).

To assess the stand-alone diagnostic value of baseline suPAR levels, C-statistics were calculated using the optimized cut-off. As shown in Figure 1, the area under the curve (AUC) regarding the diagnosis of AMI was 0.57. According to Youden-index, the optimized cut-off for suPAR for discrimination between AMI and non-AMI was 3.7 ng/mL.

Table 2 shows the accuracy for the rule-out of non-AMI using troponin levels at baseline, troponin levels at hour 1, electrocardiogram (ECG) at admission, and suPAR levels at baseline. The rationale for variations of rule-out testing has been described elsewhere [13]. Addition of suPAR reduced the number of patients who were correctly ruled-out as non-AMI: 248 patients were ruled out according to the baseline hs-TnI levels. Adding baseline suPAR on top of hs-TnI would have ruled out only 195 of the 248 patients (*p* < 0.001). In line with this finding, 454 patients were ruled out according to baseline and hour 1 troponin values. Adding baseline suPAR on top of hs-TnI, would have ruled out only 327 of the 454 patients (*p* < 0.001). Using suPAR at a cut-off of 3.7 ng/mL alone would have correctly ruled-out 58.6% of non-AMI patients, while falsely 47.3% of AMI patients would have been ruled-out.

Table 3 gives the sensitivity and specificity for the rule-out of non-AMI using troponin levels at baseline, troponin levels at hour 1, ECG at admission, and suPAR levels at baseline. Both measures do not improve upon the inclusion of suPAR.

Table 4 shows the accuracy for the rule-in of AMI using troponin levels at baseline, troponin levels at hour 1, and suPAR levels at baseline. The rationale for variations of rule-in testing has been described elsewhere [13]. Addition of suPAR reduced the number of patients, who were correctly ruled-in as AMI: 150 patients were ruled in according to hs-TnI levels. Adding baseline suPAR on top of hs-TnI, would have ruled in only 76 of the 150 patients (*p* < 0.001). Using suPAR at a cut-off of 3.7 ng/mL alone would have correctly ruled-in 52.7% of AMI patients, while falsely 41.4% of non-AMI patients would have been ruled-in.

Table 5 gives the sensitivity and specificity for the rule-in of non-AMI using troponin levels at baseline, troponin levels at hour 1, ECG at admission, and suPAR levels at baseline. Both measures do not improve upon the inclusion of suPAR.

Table 6 gives the prognostic accuracy of the rule-out definition using troponin levels at baseline, troponin levels at hour 1, ECG at admission, and suPAR levels at baseline. As shown, suPAR did not improve prognosis.

Table 7 gives the prognostic accuracy of the rule-in definition using troponin levels at baseline, troponin levels at hour 1, and suPAR levels at baseline. As shown, suPAR again did not improve prognosis.

## 4. Discussion

In this study, we evaluated the incremental diagnostic value of suPAR on top of hs-TnI in AMI. SuPAR failed in improvement of rule-out and rule-in of patients with suspected AMI. Moreover, suPAR–both in the rule-out and in the rule-in definition did not improve the 12-months prognosis regarding the endpoint’s mortality, incident non-fatal AMI, coronary intervention, and cardiac rehospitalization.

We report three major findings: first, with median suPAR levels of 3.8 ng/mL in AMI patients, and 3.3 ng/mL in patients with non-AMI suPAR levels were higher in myocardial infarction.

Second, stand-alone suPAR at admission failed regarding the diagnosis of AMI. As shown in C-statistics (at an optimized cut-off of 3.7 ng/mL), the AUC was 0.57 only.

Third, the combination of suPAR and serial hs-TnI did not improve the diagnosis of AMI as compared with hs-TnI alone. Baseline suPAR levels on top of troponin values at admission and hour 1 reduced the number of patients, who were correctly ruled-out as non-AMI, and who were correctly ruled-in as AMI.

### 4.1. SuPAR for the Diagnosis of AMI

Biomarkers are critical instruments in terms of risk prediction. Emerging data proves that new pathways and pathophysiological hypotheses yield biomarkers beyond the established ones [20,21,22,23,24]. The failure of suPAR in the diagnosis of AMI seems surprising given the results of previous smaller studies [11,12]. Both studies did not report the diagnostic value of suPAR, but rather its prognostic value in AMI patients. With a median 3.8 ng/mL in AMI patients and 3.3 ng/mL in patients with non-AMI suPAR levels seem in range with current references [9,10]. Nevertheless, the two previous reports in patients with ACS report clearly higher values [11,12]. The early Danish report from 2013 in 449 consecutive chest pain patients in a single-center yielded a median suPAR level of 5.80 ng/mL for fatal AMI, and 4.44 for non-fatal AMI using the same assay for suPAR measurement as in our study. In line with this finding, median maximum hs-TnT level in the Danish study was 149 ng/L. Similarly, in a recent Austrian case-cohort study (*n* = 194), serum suPAR levels were 29% higher in the 57 AMI (NSTEMI) patients (our study: 13%), compared with the 76 healthy controls. Again, hs-Tn levels were relatively high, at a mean of 986.3 ng/L in AMI, and the rate of subjects diagnosed with AMI was 74% compared with only 20% in our study. Taken together, one may hypothesize, that our cohort represents a relative low-risk cohort, and that incremental value of suPAR on top of hs-Tn might only evolve in mid-and high-risk cohorts.

### 4.2. Strengths and Limitations

Strengths of this study include the large cohort size and the comprehensive pre-analytics. However, like in most reported studies of patients with suspected AMI, women were under-represented, and future studies should address sex-specific differences in suPAR related diagnostic accuracy.

## 5. Conclusions

Our study shows that circulating levels of suPAR on top of hs-TnI do not improve the early diagnosis of AMI.

## Figures and Tables

**Table 1:** Characteristics of the study cohort at baseline.

	All	Non-AMI	AMI	*p*-Value
Number of patients	1220	975	245	
Age (years)	65.0 (51.0, 75.0)	63.0 (50.0, 74.0)	70.0 (60.0, 77.3)	<0.001
Male No. (%)	774 (63.4)	615 (63.1)	159 (64.9)	0.65
BMI (kg/m^2^)	26.1 (23.6, 29.4)	26.0 (23.6, 29.3)	26.6 (23.6, 29.8)	0.34
Hypertension No. (%)	825 (67.8)	631 (64.9)	194 (79.5)	<0.001
Hyperlipoproteinemia No. (%)	464 (38.0)	345 (35.4)	119 (48.6)	<0.001
Diabetes No. (%)	160 (13.3)	118 (12.3)	42 (17.2)	0.055
Former smoker No. (%)	370 (30.4)	291 (29.9)	79 (32.2)	0.53
Current smoker No. (%)	297 (24.4)	233 (24.0)	64 (26.1)	0.54
History of CAD (%)	417 (34.2)	310 (31.8)	107 (43.7)	<0.001
eGFR (mL/min for 1.73 m^2^)	76.7 (59.2, 92.3)	79.4 (61.0, 93.9)	67.4 (51.8, 82.5)	<0.001
Chest pain onset ≥ 6 h No. (%)	645 (57.4)	515 (57.8)	130 (56.0)	0.68
Troponin I 0 h (ng/L)	6.5 (3.0, 17.8)	5.1 (2.5, 10.3)	71.7 (16.2, 695.0)	<0.001
Troponin I 1 h (ng/L)	6.8 (3.1, 21.8)	5.1 (2.6, 10.5)	137.8 (33.7, 754.1)	<0.001
suPAR 0 h (ng/mL)	3.4 (2.4, 4.9)	3.3 (2.3, 4.7)	3.8 (2.6, 5.4)	0.001

AMI = acute myocardial infarction, BMI = body mass index, CAD = coronary artery disease; For continuous variables, median (25th percentile, 75th percentile) is shown. For binary variables, percentage is given.

**Table 2:** Performance in rule-out using biomarkers and ECG.

	Rule-Out Observations:		
	All (*n* = 1220)	Non-AMI (*n* = 975)	AMI (*n* = 245)
hs-TnI 0 h ≤ 3 ng/L AND low risk ECG	248 (20.7%)	248 (25.9%)	0 (0%)
hs-TnI 0 h ≤ 3 ng/L AND low risk ECG AND suPAR 0 h < 3.7 ng/mL	195 (16.2%)	195 (20.4%)	0 (0%)
hs-TnI 0 h ≤ 6 ng/L AND hs-TnI 1 h ≤ 6 ng/L	454 (38.1%)	450 (47.4%)	4 (1.6%)
hs-TnI 0 h ≤ 6 ng/L AND hs-TnI 1 h ≤ 6 ng/L AND suPAR 0 h < 3.7 ng/mL	327 (27.4%)	325 (34.2%)	2 (0.8%)
suPAR 0 h < 3.7 ng/mL	687 (56.3%)	571 (58.6%)	116 (47.3%)

ECG = electrocardiogram, AMI = acute myocardial infarction, hs-TnI = high-sensitivity troponin I, suPAR = soluble urokinase-type plasminogen activator receptor.

**Table 3:** Sensitivity analyses in rule-out using biomarkers and ECG.

	NPV	Sensitivity	PPV	Specificity
hs-TnI 0 h ≤ 3 ng/L AND low risk ECG	100 (98.5, 100)	100 (98.5, 100)	25.4 (22.7, 28.3)	25.9 (23.1, 28.8)
hs-TnI 0 h ≤ 3 ng/L AND low risk ECG AND suPAR 0 h < 3.7 ng/mL	100(98.1, 100)	100 (98.5, 100)	24.1 (21.5, 26.8)	20.4 (17.8, 23)
hs-TnI 0 h ≤ 6 ng/L AND hs-TnI 1 h ≤ 6 ng/L	99.1 (97.8, 99.8)	98.4 (95.9, 99.6)	32.5 (29.1, 36.0)	47.4 (44.2, 50.7)
hs-TnI 0 h ≤ 6 ng/L AND hs-TnI 1 h ≤ 6 ng/L AND suPAR 0 h < 3.7 ng/mL	99.4 (97.8, 99.9)	99.2 (97.1, 99.9)	27.9 (25.0, 31.1)	34.2 (31.2, 37.4)
suPAR 0 h < 3.7 ng/mL	83.1 (80.1, 85.8)	52.7 (46.2, 59.0)	24.2 (20.6, 28.1)	58.6 (55.4, 61.7)

**Table 4:** Performance in rule-in using biomarkers and ECG.

	Rule-In Observations:		
	All (*n* = 1220)	Non-AMI (*n* = 975)	AMI (*n* = 245)
hs-TnI 1 h > 6 ng/L AND hs-TnI increase ≥ 12 ng/L compared to hs-TnI 0 h	177 (15.3%)	27 (2.9%)	150 (65.5%)
hs-TnI 1 h > 6 ng/L AND hs-TnI increase ≥ 12 ng/L compared to hs-TnI 0 h AND suPAR 0 h ≥ 3.7 ng/mL	93 (8.1%)	17 (1.8%)	76 (33.2%)
suPAR 0 h ≥ 3.7 ng/mL	533 (43.7%)	404 (41.4%)	129 (52.7%)

AMI = acute myocardial infarction, hs-TnI = high-sensitivity troponin I, suPAR = soluble urokinase-type plasminogen activator receptor.

**Table 5:** Sensitivity analyses in rule-in using biomarkers and ECG.

	NPV	Sensitivity	PPV	Specificity
hs-TnI 1 h > 6 ng/L AND hs-TnI increase ≥ 12 ng/L compared to hs-TnI 0 h	91.9 (90.0, 93.6)	65.5 (59.0, 71.6)	84.7 (78.6, 89.7)	97.1 (95.8, 98.1)
hs-TnI 1 h > 6 ng/L AND hs-TnI increase ≥ 12 ng/L compared to hs-TnI 0 h AND suPAR 0 h ≥ 3.7 ng/mL	85.6 (83.3, 87.7)	33.2 (27.1, 39.7)	81.7 (72.4, 89.0)	98.2 (97.1, 98.9)
suPAR 0 h ≥ 3.7 ng/mL	83.1 (80.1, 85.8)	52.7 (46.2, 59.0)	24.2 (20.6, 28.1)	58.6 (55.4, 61.7)

**Table 6:** Prognostic value of suPAR within the rule-out definition.

RULE-OUT-Definition	Death	Incident Non-Fatal AMI	Coronary Intervention	Cardiac Rehospitalisation
hs-TnI 0 h ≤ 3 ng/L AND low risk ECG	*N* = 0/246 0%	*N* = 0/246 0%	*N* = 5/246 2.12%	*N* = 20/246 8.96%
hs-TnI 0 h ≤ 3 ng/L AND low risk ECG AND suPAR 0 h < 3.7 ng/mL	*N* = 0/193 0%	*N* = 0/193 0%	*N* = 3/193 1.55%	*N* = 12/193 6.75%
hs-TnI 0 h ≤6 ng/L AND hs-TnI 1 h ≤ 6 ng/L	*N* = 6/452 1.46%	*N* = 3/452 0.75%	*N* = 11/452 2.55%	*N* = 52/452 12.50%
hs-TnI 0 h ≤ 6 ng/L AND hs-TnI 1 h ≤6 ng/L AND suPAR 0 h < 3.7 ng/mL	*N* = 2/325 0.63%	*N* = 0/325 0%	*N* = 8/325 2.54%	*N* = 30/325 9.83%
suPAR 0 h < 3.7 ng/mL	*N* = 10/685 1.71%	*N* = 4/685 0.71%	*N* = 27/685 4.01%	*N* = 116/685 18.16%

AMI = acute myocardial infarction, hs-TnI = high-sensitivity troponin I, suPAR = soluble urokinase-type plasminogen activator receptor.

**Table 7:** Prognostic value of suPAR within the rule-in definition.

RULE-IN-Definition	Death	Incident Non-Fatal AMI	Coronary Intervention	Cardiac Rehospitalisation
hs-TnI 1 h > 6 ng/L AND hs-TnI increase ≥ 12 ng/L compared to hs-TnI 0h	*N* = 11/177 6.78%	*N* = 3/177 2.08%	*N* = 16/177 9.57%	*N* = 46/177 28.82%
hs-TnI 1 h > 6 ng/L AND hs-TnI increase ≥ 12 ng/L compared to hs-TnI 0 h AND suPAR 0 h ≥ 3.7 ng/mL	*N* = 9/93 10.28%	*N* = 2/93 3.03%	*N* = 8/93 9.36%	*N* = 23/93 28.00%
suPAR 0 h ≥ 3.7 ng/mL	*N* = 44/533 9.91%	*N* = 7/533 1.66%	*N* = 28/533 5.83%	*N* = 120/533 26.60%

AMI = acute myocardial infarction, hs-TnI = high-sensitivity troponin I, suPAR = soluble urokinase-type plasminogen activator receptor.
